# Does chronic lymphocytic thyroiditis influence the staging of differentiated thyroid carcinoma?

**DOI:** 10.1590/S1808-86942011000100013

**Published:** 2015-10-19

**Authors:** Marcos Antonio Nemetz, Fúlvio Clemo Santos Thomazelli, Luzete Cristina Silva Granero, Ana Beduschi Nemetz, Marcela Bonalumi dos Santos

**Affiliations:** 1 MSc, Professor; 2 MSc in Medical Sciences - Federal University of Rio Grande do Sul - UFRGS, Endocrinologist. Professor of Endocrinology - Curso de Medicina da Fundação Universidade Regional de Blumenau; 3 MSc in Medical Sciences - Federal University of São Paulo - UNIFESP, Pathologist; 4 Medical student - Fundação Universidade Regional de Blumenau; 5 Medical student - Fundação Universidade Regional de Blumenau

**Keywords:** thyroidectomy, thyroiditis, thyroid neoplasms, neoplasm staging

## Abstract

The association between differentiated thyroid carcinoma (DTC) and chronic lymphocytic thyroiditis (CLT) has been reported in literature.

**Aim:** To evaluate the incidence of this association and to determine whether the CLT may influence on the early initial staging of DTC when associated with other variable risks.

**Study design:** Historical (retrospective) cohort.

**Materials and Methods:** Fifty two patients with DTC were evaluated from 1999 to 2009. They were divided into two groups. The first group had 35 patients with DTC without DLT; the second had 17 patients with CLT. Total thyroidectomy was the treatment chosen for all patients. Similarities shared in both groups such as age, gender, histological tumor type, tumor diameter, regional only or with distant metastases, extrathyroidal invasion, multifocality and presence of tumor capsule were considered. T-Student tests and Chi-square tests were applied to analyze the data.

**Results:** The incidence of DTC without CLT was higher that of DTC+CLT (p=0.0126). We noticed no statistic differences between the common variables analyzed.

**Conclusions:** CLT occurred in 33% of the patients with DCT. All cases of DTC were in the early stages.

## INTRODUCTION

Thyroid cancer is responsible for about 1% of the new cases of diagnosed malignant diseases^1^; and is divided according to histological type into papilliferous, follicular, undifferentiated, a little differentiated and malignant lymphoma, among others^2^. papilliferous and the follicular carcinoma are also called thyroid differentiated carcinoma (TDC), although they have distinct clinical, histological and biological patterns^3^. They correspond to approximately 90% of the cases of malignant neoplasia of the thyroid^1,^^4^. When diagnosed early on, TDC is a tumor which is usually curable^5^. Studies point out that these cancers have a cure rate of approximately 80%, while 20% will have local recurrence and 5 to 10% will develop distant metastasis^4^.

Besides the classical form, the papilliferous cancers have distinct histological variants identified as: follicular, trabecular, insular, solid, of high cells, of columnar cells, diffuse sclerosing, oxyphilic, amongst others also reported in the literature^2,^^6^. These patterns, despite controversial and still not standardized, are not necessarily exclusive, since one single tumor may fit the description of multiple variants^2^. The majority has only morphological differences, and it does not always have significant prognostic value^2^. The follicular carcinoma is further divided according to invasion and growth patterns^2,^^6^. As to invasion, we know two forms: minimally invasive and broadly or largely invasive^2,^^6^. The growth pattern may vary from one well differentiated form to another which is not so well differentiated^2,^^6^.

The TDC patient may be classified into high, low and very low risk^1^. When analyzing the initial tumor, it is very important to know what is its likely prognosis^7^. In recent years, the search for factors which can influence prognosis has been a constant, since they impact directly on the identification of patients who have a more aggressive disease, as well as its treatment and follow up^8^. Multiple series of cases have been assessed and numerous staging classifications have been proposed taking into account risk and prognostic factors (TNM, EORTC, UICC/AJCC, AGES, AMES, MACIS, Clinical classification, Ohio)^3,^^9^, ^10^, ^11^, ^12^, ^13^, ^14^, ^15^, ^16^. Nonetheless, the relative impact of each one of these indicators is yet to be clearly defined.

Many reports have stressed the concurrence between chronic lymphocytic thyroiditis (CLT) and TDC, which has been pointed as a prognostic factor for this type of neoplasia^17^, ^18^, ^19^, ^19^, ^20^, ^21^. CLT is a disorder stemming from the fact that T suppressor lymphocytes are rendered incapable of destroying clones of lymphocytes sensitized by thyroid antigens, with consequent cytotoxicity mediated by natural killer cells and the interaction of T-Helper lymphocytes with lymphocytes B, producing antibodies against thyroid components^2^.

Studies have investigated the possible relationship between the chronic lymphocytic infiltrate expression and the degree of histological aggressiveness in thyroid tumors^18,^^19,^^21,^^22^. Notwithstanding, most of these papers assessed only the lymphocitic thyroid disease associated with well differentiated carcinoma in general, without investigating whether these lymphocytic infiltrates, when associated with the other risk factors, would be associated to low or high risk. The goal of the present paper was to establish the relative frequency of CLT in patients with TDC, and whether the CLT in a multivariate risk analysis could influence tumor staging and, indirectly, the prognosis of this disease.

## MATERIALS AND METHODS

We carried out a historical cohort study (retrospective), encompassing a period of 10 years - between July of 1999 and May of 2009, when we analyzed the pathology reports from the medical charts of 54 patients diagnosed with TDC who were submitted to total thyroidectomy, from whom we had the following data: gender, age upon diagnosis, tumor diameter, tumor capsule findings, thyroid invasion, multifocality, locoregional metastasis, histology pattern, chronic lymphocitic thyroiditis and distant metastasis; and these were the inclusion criteria for the present study. Two charts were excluded for not having complete data. The material utilized was obtained from files from the Private Clinic and the Pathology Lab.

These charts and reports were separated in two groups: the first with TDC diagnosis, and the second were those with TDC associated with CLT. All the patients were submitted to total thyroidectomy, with or without neck lymph node dissection, and radiotherapy. Neck dissection was indicated for those patients who had clinical signs of lymph node involvement, found in pre-op ultrasound exam or during surgery.

TDC diagnosis and that of its variants was based on the criteria defined by the World Health Organization in 2004, later modified^2,^^6^. CLT was defined as the presence of lymphocitic infiltrate, varying from islands in the tumor itself or around it, all the way to large infiltrates, also including germinative centers^23^. Patients with the classic Hashimoto's Thyroiditis (HT) were also included in the study as having lymphocitic thyroiditis.

The material obtained from the thyroidectomies was analyzed by 4 pathologists of the same team, by means of macroscopic and microscopic assessments. The later by means of paraffin block slides, in histologic cross-sections dyed by the hematoxylin-eosin technique, using the electronic microscope.

The distant metastasis variable was assessed after surgery by means of a full body scan with radioactive iodine.

Tumor staging (Table 1) and risk stratification (Table 2) were assessed in accordance with the classification from the *American Joint Committee on Cancer* (AJCC) / Tumor *Nodes Metastasis* (TNM)^24,^^25^.

**Table 1:** TNM classification for thyroid cancer

T (TUMOR)	N (LYMPH NODE METASTASES)	M (DISTANT METASTASES)
T1 £ 2 cm (T1a £ 1cm T1b 1-2 cm)	N0 absent	M0 absent
T2 2-4 cm	N1a metastases at VI level	M1 distant metastases
T3 > 4cm confined to the thyroid or with minimum extrathyroid invasion	N1b neck metastases (lateral) or in the upper mediastinum	
T4a invading subcutaneous tissue, larynx, trachea, esophagus, or laryngeal recurrent		
T4b invading pre-vertebral fascia or involving the carotid or mediastinal vessels		
Tx unknown size without extrathyroid invasion	Nx lymph nodes not assessed	Mx not assessed

TNM: Classification proposed by the American Joint Committee on Cancer (2002).

**Table 2:** Staging of undifferentiated thyroid tumors.

Stage	< 45 years	^3^ 45 years
Papillary or follicular carcinoma		
I	Any T, any N, M0	T1, N0, M0
II	Any T, any N, M1	T2, N0, M0
III	-	T3, N0, M0
T1/T2/T3, N1a, M0		
IV A	-	T4a, any N, M0
T1/T2/T3, N1b, M0		
IV B	-	T4b, any N, M0
IV C	-	any T, any N, M1

Classification proposed by the American Joint Committee on Cancer (2002).

Variables common to groups TDC and TDC + CLT were statistically analyzed in order to look for differences between them.

For database management, statistical calculations and to create the tables and graphs we used Microsoft Excel^®^ version 2003. The t Student test was used in order to compare the groups as the quantitative variables age and tumor diameter. In order to associated the qualitative variables: gender, extrathyroid invasion, tumor capsule, multifocality, distant metastasis, locoregional metastasis, histological pattern and tumor staging we used the chi-squared test. In all the statistical tests we used the 5% (p<0,05) significance level.

This study was approved by the Ethics in Research Committee of the Institution, under protocol # 057/09.

## RESULTS

All the charts were from patients with papilliferous carcinoma. Of these, 17 (33%) had TDC together with CLT, and 35 (67%) had TDC without CLT (p=0.01) (Table 3). We found no case of follicular carcinoma.

**Table 3:** Patient distribution according to the presence of CLT (chronic lymphocitic thyroiditis).

Presence of CLT (chronic lymphocitic thyroiditis)	Number of patients	%	CI (95%)
Yes	17	32,69	(19,94 - 45,44)
No	35	67,31	(54,56 - 80,06)
Total	52	100,00	

(c^2^ = 6,23; gl = 1; *p* = 0,0126)

The ages varied between 14 and 80 years, with a mean of 45.9 years. There was one case of TDC in a 14-year-old girl. In the TDC+CLT group, the youngest patient was a 22 year-old woman. There was no significant difference between the groups as far as age is concerned (Table 4). In the TDC group, tumor diameter varied between 0.3 and 6 cm, in the TDC_CLT group, tumor size was smaller - 0.4 and 3.5 cm (*p*=0.8) (Table 4).

**Table 4:** Comparing the groups in relation to patient age and tumor diameter.

Group age(years) Diameter(cm)			
	N	Mean	Mean
TDC	35	43;1 (14-80)	1,4(0,3-6)
TDC + CLT	17	51;3(22-76)	1,3(0,4-3,5)
Total	52	45;9(14-80)	1,33(0,3-6)

Age: (*p* = 0.1); Tumor diameter: (*p* = 0.8794). Comparison through the t Student test.

CLT: chronic lymphocitic thyroiditis; TDC: thyroid differentiated carcinoma; TDC+CLT: thyroid differentiated carcinoma associated with chronic lymphocitic thyroiditis.

The microcarcinoma (tumor smaller than 1cm), was most frequently found in the TDC+CLT group, when compared to the TDC group: 11 (64.7%) and 18 (51.4%), respectively (*p*=0.5) (Table 5).

**Table 5:** Distribution of the patients with TDC (thyroid differentiated carcinoma) according to microcarcinoma and the presence of CLT (chronic lymphocitic thyroiditis).

Microcarcinoma	CLT		Total
	No	Yes	
No	17 (48,6%)	6 (35,3%)	23 (44,2%)
Yes	18 (51,4%)	11 (64,7%)	29 (55,8%)
Total	35 (100,0%)	17 (100,0%)	52 (100,0%

(c^2^ = 0,37; gl = 1; *p* = 0,54407)

The results from the multivariate analysis are depicted on Table 6.

**Table 6:** Comparing the TDC and TDC+CLT groups in relation to tumor variables and patient variables.

Tumor variables		TDC n (%)	TDC + CLT n (%)	TOTAL n (%)	Comparing TDC with and without CLT (p)*
	£ 2 cm	28 (80%)	12 (70,6%)	40 (76,9%)	0,48878
Tumor diameter	> 2 e £ 4 cm	6 (17,1%)	5 (29,4%)	11 (21,2%)	
	> 4 cm	1 (2,9%)	0 (0%)	1 (1,9%)	
Tumor capsule	Absent	22 (62,9%)	12 (70,6%)	34 (65,4%)	0,58253
	Present	13 (37,1%)	5 (29,4%)	18 (34,6%)	
Multifocality	No	25 (71,4%)	9 (52,9%)	34 (65,4%)	0,31548
	Yes	10 (28,6%)	8 (47,1%)	18 (34,6%)	
Extrathyroid invasion	No Yes	29 (82,9%) 6 (17,1%)	13 (76,5%) 4 (23,5%)	42 (80,8%) 10 (19,2%)	0,86257
Distant metastasis	No	35 (100%)	17 (100%)	52 (100%)	-
	Yes	-	-	-	
Locoregional metastasis	No	28 (80%)	14 (82,4%)	42 (80,8%)	0,86257
	Yes	7 (20%)	3 (17,7%)	10 (19,2%)	
	Papilliferous Classic	25 (71,4%)	14 (82,4%)	39 (75%)	0,60943
Histology pattern	Papilliferous follicular variant	9 (25,7%)	3 (17,7%)	12 (23,1%)	
	Papilliferous oxyphilic variant	1 (2,9%)	0 (0%)	1 (1,9%)	
Patient variables					
Gender	Females	32 (91,4%)	16 (94,1%)	48 (92,3%)	0,83105
	Males	3 (8,6%)	1 (5,9%)	4 (7,7%)	
Age in years	< 45 years	17 (48,6%)	4 (23,5%)	21 (40,4%)	0,15412
	^3^ 45 years	18 (51,4%)	13 (76,5%)	31 (59,6%)	

*Association between the variables done through the chi-squared test. TDC: thyroid differentiated carcinoma. TDC + CLT: thyroid differentiated carcinoma associated with chronic lymphocitic thyroiditis.

The distribution by tumor diameter, within each group was: 28 cases (80%) smaller than or equal to 2 cm in the TDC group, and 12 (70.6%) in the TDC + CLT; 6 cases (17.1%) larger than 2cm and smaller than or equal to 4cm in the TDC group, and 5 (29.5%) in the TDC + CLT. There was only one case in which the tumor was larger than 4cm, it happened in the TDC group. There were no significant differences between patients in the TDC group in relation to the greater tumor diameter.

In the TDC and TDC+CLT groups there was a difference which was only proportional to the presence or absence of the capsule. In the TDC group, 37.1% of the patients had the capsule, while in the TDC+CLT group, 29.4% only had a capsule (*p*=0.5).

We can notice a predominance of multifocality in the TDC+CLT group, however such difference was not statistically significant.

In the patients with TDC+CLT, we noticed a higher frequency of extrathyroid invasion (17.1% vs. 23.5%), however with a lower rate of locoregional metastasis (17.7% vs. 20%). No patient in this sample had distant metastasis.

As to carcinoma distribution according to histology, 39 cases (75%) were classic TDC, and 13 cases were its variants. We also noticed a predominance of the follicular variant, 12 cases (23.1%), and there was only one case (1.9%) of the oxyphilic cells variant, which occurred in the TDC group (*p*=0.6) (Table 6).

There was a predominance of females, 48 cases (92.3%), and 4 cases (7.7%) of males. In the TDC group, 32 (91.4%) patients were females, and in the TDC+CLT, 16 (94.1%) were females and only 1(5.9%) patient was male (*p*=0.8).

In both groups the initial stages I and II were predominant, without considerable statistical difference (Table 7).

**Table 7:** TNM in patients with TDC with and without CLT.

	TDC with CLT (n = 17)	TDC without CLT (n = 35)	P
TNM classification			
Stage I	11 (26,8%)	30 (73,2%)	
Stage II	2 (66,7%)	1 (33,3%)	NS
Stage III	1 (50%)	1 (50%)	
Stage IV a	3 (50%)	3 (50%)	

TDC: thyroid differentiated carcinoma; CLT: chronic lymphocitic thyroiditis, P = NS: statistically non-significant, TNM: Classification proposed by the American Joint Committee on Cancer (2002).

## DISCUSSION

Since its first publication, the TDC and CLT relation is controversial, and it has been largely discussed in the literature, with results varying between those which did not prove the association^26^ and others in which it is clear^17^, ^18^, ^19^, ^20^, ^21^,^27^. In many of these series, the papilliferous carcinoma was the predominant histological pattern, and this was also true in the present paper. In 32.69% of the cases there was an association between the CLT and the TDC, which coincides with data reported in the literature.

It is uncertain whether the coexisting thyroiditis could be the cause or consequence of the thyroid carcinoma, and whether it influences its staging and its prognosis indirectly.

It is believed that CLT induces the malignant transformation because it stimulates mitosis and follicular epithelium proliferation through cell necrosis, caused by the chronic infiltration of lymphocytes and hormonal thyroid stimulation, or by a partial disorganization of the antitumor autoimmune surveillance^18^. On the other hand, some authors consider the lymphocytic infiltration process as benign; thus inferring that CLT would not be a premalignant lesion, but rather an autoimmune mechanism involved in the TDC^28^. It has been reported that the TDC has a better prognosis when associated with the CLT^29^.

In this analysis we did not find significant differences between the TDC and TDC+CLT groups in relation to tumor diameter, although there are papers proving the CLT association to initial stage tumors^30^. Other authors also did not find significant associations comparing multifocality, age and gender between patients with and without lymphocytic infiltrates^27^. One study reported that 30% of the patients with thyroid papilliferous carcinoma had coexisting CLT. However, the better prognosis reported would be more related to a minimum protective effect, since it is more common to young female patients^31^. Papers have shown a lower incidence of distant metastasis and disease recurrence in patients with associated CLT^29^.

Better prognoses are: those microcarcinoma or encapsulated tumors: the classic and follicular variants are of medium prognosis and the high cells and column cells are those considered the worst prognosis^8^. Although the results were not statistically significant, in the present study we found 64.7% microcarcinomas in the TDC+CLT group vs. 51.4% in the TDC group.

Although the CLT and TDC association has been broadly proven in many papers, the following aspects remain uncertain:

1. Do these two thyroid disorders share a common etiology?
2. Does the CLT affect the TDC biological behavior?
3. Do the chronic lymphocitic infiltrates represent a host immune response to the tumor or is it an isolate occurrence?

Studies with larger samples and with long term follow up are still necessary in order to clear up these issues.

Nonetheless, these authors believe that the TDC+CLT cases are of low risk and good prognosis, since most of these patients were staged as initial, implying a possible protective role of the lymphocitic infiltrate.

## CONCLUSION

1. CLT associated with the TDC happened in 17 (33%) of the 52 patients with differentiated carcinomas of the thyroid.
2. The CLT did not influence tumor staging in patients with TDC.
3. There were no statistical differences between the variables: age, gender, histology pattern, tumor diameter, locoregional or distant metastasis, extrathyroid invasion, multifocality, and the presence of tumor capsule in the groups with and without CLT.
4. Considering the risk factors all together, most of the TDC+CLT cases were staged as initial.
